# Trends in contraceptive practices among women in reproductive age at a health facility in Ghana: 2011–2013

**DOI:** 10.1186/s40834-016-0010-9

**Published:** 2016-02-23

**Authors:** Hubert Amu, Samuel H. Nyarko

**Affiliations:** 1 Department of Population and Health, University of Cape Coast, Cape Coast, Ghana; 2 Department of Population and Behavioural Sciences, School of Public Health, University of Health and Allied Sciences, Hohoe, Ghana

**Keywords:** Trends, contraceptive, reproductive age, health facility, Ghana

## Abstract

**Background:**

There is dearth of information on contraceptive use among women in reproductive age in Ghana over time. This study sought to examine the trends in contraceptive practices among women in reproductive age in a health facility in Ghana in terms of acceptor rates, age at first reporting and couple-years of protection.

**Methods:**

The contraceptive data of women were extracted from the registry of a health facility for a three-year period from 2011 to 2013. Graphs and tables were used to present the trends in the use of various contraceptive methods for the study period.

**Results:**

Depot Medroxyprogesterone Acetate (Depo-Provera) was the most accepted contraceptive method as well as the most protective method against unwanted pregnancies for the three-year period. However, male condom, estradiol valerate/norethindrone enanthate (Norigynon) and pills were the lowest among all the contraceptives used by women for the study period in terms of acceptance, while intra-uterine device had the lowest couple-years of protection.

**Conclusions:**

Some contraceptive methods have been consistently under-utilised by women in the catchment area and needed to be promoted to improve upon the contraceptive use rate.

## Background

Globally, contraceptive practice has improved over the past three decades [1–5]. According to Guttmacher Institute [5], in developed countries, about 62 % of all women in reproductive age practice contraception. In sub-Saharan Africa, it is estimated that 14 million unintended pregnancies occur every year, with almost half occurring among women aged 15–24 [6]. Risk of exposure to pregnancy has increased over the past three decades with a widening gap between sexual debut and age of marriage and has therefore increased sexual activity prior to marriage [7, 8], placing women of reproductive age at increased risk of pregnancy, especially when they are most socially and economically vulnerable [9, 10].

In Ghana, the 2008 Ghana Demographic and Health Survey (GDHS) showed an increase in contraceptive use among currently married women in Ghana from 13.0 % in 1988 to 23.5 % in 2008. Similarly, an increase in the proportion of sexually active unmarried women using a contraceptive method was noted to rise from 43.5 to 50.4 % between 2003 and 2008 [11]. However, even though about seventy-five percent of women in reproductive age reported having had sex by age twenty [12, 13], the contraceptive prevalence rate of the country was only 23.4 % as indicated by the 2010 Population and Housing Census of Ghana [14]. This is an indication that only a few of the sexually active women use contraceptives [7].

Quite a number of studies have been conducted on contraceptive practices among women in reproductive age both in and outside health facilities [5, 15–20]. In spite of this, little is known on the trends of contraceptive practices among women in reproductive age over time in the country. Consequently, this study sought to examine the trends in the use of various contraceptive methods among women in reproductive age from 2011 to 2013. The trends in the use of the contraceptives were examined in terms of the acceptor rates, age at first reporting of contraceptives and couple-years of protection for each contraception method for the study period. It is believed that this paper will help fill the literature gap on the trends of contraceptive use over time in Ghana.

## Methods

The setting of this study was the Kasoa Polyclinic in the Awutu*-*Senya*-*East District, Central Region, Ghana. The facility was established in the year 1993 as Kasoa Health Centre. It was however upgraded to the status of a polyclinic in 2013 after Kasoa became the capital of the newly created district. Kasoa Polyclinic provides 24-h in-patient, out-patient and emergency services. Maternal health services are integrated and made accessible to all women in the sub-metro within the context of Primary Health Care (PHC).

The study used data collected from the facility’s registry (contraceptive records). The target population of this study comprised women in reproductive age (15–49 years) who accessed contraceptive services at the facility’s fertility clinic. The numbers of women in reproductive age for 2011, 2012 and 2013 at the Kasoa Sub-district (the catchment area for Kasoa Polyclinic) were 15984, 24830 and 6144 respectively. Contraceptive records of these women were therefore the source of data. Institutional approval was obtained from the administration of the health facility.

A study protocol was used to collect the data from records on contraceptive practices of women from 2011 to 2013. The study protocol was based on the age, marital status, religion, acceptor rate of contraceptives and age at first reporting or use of contraceptive as well as couple-years of protection during contraceptive usage. Data on the ages of the women were collected in single years but grouped in five-year intervals for analysis.

Acceptor rate of contraceptives refers to the level of utilisation of contraceptives. It is therefore an important indicator to consider in assessing the contraceptive practices of women in reproductive age. The acceptor rate of contraceptives is expressed as a percentage of number of acceptors to total number of women in reproductive age within a given year. Mathematically, it is denoted as:

$$ \mathrm{ARC}\frac{\mathrm{Number}\kern0.5em \mathrm{of}\kern0.5em \mathrm{a}\mathrm{cceptors}\kern0.5em \mathrm{of}\kern0.5em \mathrm{contraceptives}\kern0.5em \mathrm{in}\kern0.5em \mathrm{a}\kern0.5em \mathrm{period}}{\mathrm{Number}\mathrm{s}\kern0.5em \mathrm{of}\kern0.5em \mathrm{women}\kern0.5em \mathrm{of}\kern0.5em \mathrm{reproductive}\kern0.5em \mathrm{a}\mathrm{ge}\;\mathrm{in}\kern0.5em \mathrm{the}\kern0.5em \mathrm{s}\mathrm{a}\mathrm{me}\kern0.5em \mathrm{period}}\times 100 $$

Couple-years of protection of a contraceptive represents the level of protection offered by a particular contraceptive against unintended pregnancies over a period of time, usually given in years. It is expressed in relation to a conversion factor assigned to specific contraceptives, depending on whether they are short-term or long-term methods of contraception. Mathematically, Couple-years of protection is denoted as: CYP = Number of contraceptives issued out × Conversion factor.

The conversion factors obtained from the study facility for the contraceptives were as follows: Depot Medroxyprogesterone Acetate [DMPA] (Depo-Provera) = 4, Norigynon = 12, Intra-uterine Device = 3.5, male condoms = 120, pills = 13, and Implant (Jadelle) = 3.5. [21]. Norigynon is a monthly injectable contraceptive which primarily prevents the occurrence of ovulation. Norigynon contains the generic salt – Estradiol valerate.

Data collection was manually done and relevant information from the records were therefore extracted and recorded. Regular verification of data was done and all inconsistencies were resolved. The data were processed with the Statistical Product and Service Solutions (SPSS Version 21) and Microsoft Excel. The results were presented graphically using bar chart and line graph as well as frequency tables.

## Results

### Socio-demographic characteristics of women

Socio-demographic characteristics of women including age, marital status and religion were presented in Table 1. With regard to age, in 2011, 48.9 % of women in reproductive age who registered for contraceptives were aged 25–29. Women aged 35 and above, however, had the least representation (8.2 %). In 2012, those aged 25–29 had the highest representation (59.2 %), followed by those aged 20–24 (19 %). Those aged 35 and above again recorded the least representation with 2.7 %. In 2013, women aged 25–29 still recorded the highest (37 %) with the least representation being women aged 15–19 (7.8 %).

**Table 1:** Socio-demographic characteristics of registrants

Socio-demographic characteristic	Year					
	2011		2012		2013	
	Freq	%	Freq	%	Freq	%
Age						
15 – 19	1029	9.5	610	10.9	363	7.8
20 – 24	2420	22.3	1069	19.0	1162	25.1
25 – 29	5316	48.9	3325	59.2	1714	37.0
30 – 34	1209	11.1	458	8.2	787	16.9
35 +	887	8.2	152	2.7	610	13.2
Marital status						
Never married	5452	50.2	3325	59.2	1976	42.6
Married	4762	43.8	2070	36.9	2559	55.2
Divorced	486	4.5	159	2.8	101	2.2
Separated	161	1.5	60	1.1	0	0
Religion						
Christianity	5,620	51.7	3,256	57.9	2,403	51.8
Islam	3,412	31.5	2,224	39.6	2,124	45.8
African Traditional	1,829	16.8	134	2.5	109	2.4
N	10861	100.0	5614	100.0	4636	100.0

Source: Field work, 2014

Concerning marital status, in 2011, about half (50.2 %) of the women were never married, 43.8 % were married while 1.5 % were separated. In 2012, those who had never married still had the highest representation with 59.2 %, followed by married women with 36.9 % while separated women were the least (1.1 %). In 2013, however, more than half (55.2 %) were married women while the least were divorced (2.2%). For religious affiliation, Christians were the majority through out the three-year period under review: 2011 (51.7 %), 2012 (57.9 %) and 2013 (51.8 %). Muslims, on the other hand, represented 31.4 %, 39.6 % and 45.8 % for 2011, 2012 and 2013 respectively.

### Acceptor rate of contraceptives

All modern contraceptives including both Mirena and ParaGard types of IUD were offered to women in all the three years under review. The numbers of women in reproductive age for 2011, 2012 and 2013 at the catchment area of the facility were 15984, 24830 and 6144 respectively. Based on the formula for calculating contraceptive acceptor rate and the population of women in reproductive age in each year, the contraceptive acceptor rates for the various contraceptives were derived and presented in Table 2. DMPA recorded the highest acceptor rates for the three-year period under review. Specifically, it recorded an acceptor rate of 24.8 % in 2011, which reduced to 10 % in 2012, but increased to 33.4 % in 2013. In 2011, oral contraceptive pills recorded the second highest acceptor rate of 6.3 % after DMPA with Implant (Jadelle) being the least (0.6 %). In 2012, however, Implant (Jadelle) was the second most accepted contraceptive after DMPA with 2.9 %. Male condoms, however, recorded the least acceptor rate of 0.6 % in 2012. Implant again recorded the second highest acceptor rate of 7.5 % in 2013 while pills had the least with 2.2 % of women in reproductive age.

**Table 2:** Acceptor rates of contraceptives from 2011–2013

Method	2011		2012		2013	
	Acceptors	Rate	Acceptors	Rate	Acceptors	Rate
DMPA	3977	24.8	2480	10	2051	33.4
Norigynon	280	1.8	250	1.0	206	3.4
Implant (Jadelle)	93	0.6	716	2.9	476	7.5
IUD	120	0.8	354	1.4	357	5.8
Pills	1002	0.3	303	1.2	135	2.2
Male condoms	139	0.9	167	0.6	143	2.3

Source: Field work, 2014

### Age at first reporting of contraceptive use

**Fig. 1:**
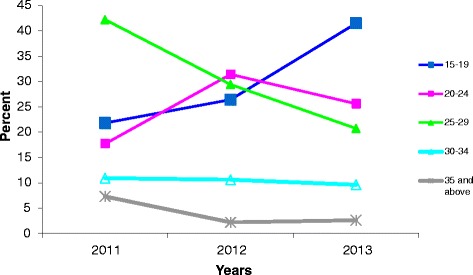
Age at first reporting of contraceptive use. Source: Field work, 2014

The study also determined the age at first reporting of contraceptive use among women in reproductive age. From Fig. 1, 42.2 % of the women first reported utilisation of contraceptive when they were age 25 to 29. This was followed by those who started using contraceptive at ages 15 to 19 (21.8 %). Only 7.3 % of the women, however, first reported the use of contraceptive at age 35 and above. In 2012, however, 31.4 % started using contraceptive at ages 20 to 24. In 2013, 41 % of the women started using contraceptive at age 15 to 19.

### Couple-years of protection of contraceptives

**Fig. 2:**
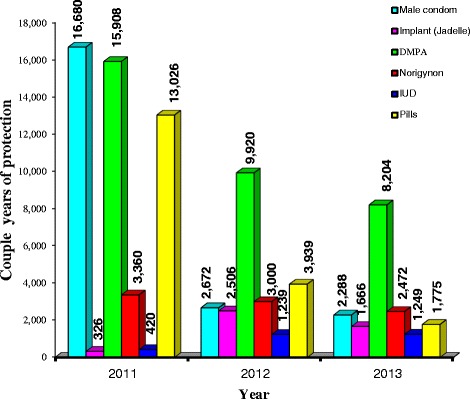
Couple-Years of Protection of Contraceptives. Source: Field work, 2014

Based on the formulae for calculating couple-years of protection, the couple-years of protection offered by contraceptives issued at the health facility from 2011 to 2013 were calculated. As indicated in Fig. 2, the highest couple-years of protection against unintended pregnancies in 2011 was provided by male condoms (16,680), followed by DMPA with 15,908. Implants (Jadelle) had the lowest number of couple-years of protection in 2011 (326). DMPA had the highest couple-years of protection in 2012 (9,920), followed by oral contraceptive pills with 3,939. The IUD had the lowest number of couple-years of protection in 2012 (1,239). DMPA again had the highest couple-years of protection in 2013 with 8,204, followed by Norigynon with 2,249 while IUD again had the lowest number of couple-years of protection (1,249).

## Discussion

DMPA was found to be the most accepted contraceptive among the women for the three-year period. This could be due to the fact that it is a medium term contraceptive and the women did not need to use them every day or anytime they wanted to have sexual intercourse as in the case of condoms and oral contraceptive pills. Also, the women might not have wanted to reveal to their husbands or partners the fact that they used contraceptives. DMPA therefore became an easily acceptable choice of contraceptive. This is consistent with findings by Okpani and Kua [22] in which they indicated that DMPA had the highest acceptor rates among a population of 1,541 women studied. On the contrary, male condom was found to have the lowest acceptor rate among the women who utilised contraceptives over the period under review. This could be due to the fact that male condom use is the preserve of men and in Ghana, it may not be morally right for women to carry male condoms with them and give them to their partners to use.

IUDs and implants were found to have been under-utilized by the women. This could be attributed to some myths about the methods among Ghanaians. Some women for instance do not want to use the IUD because they believe that the IUD can cause infection or that it can travel from the uterus to other parts of the body [23]. Some also believe that the IUD prevents pregnancy by causing abortions. Some women also believe that the IUD causes infertility and ectopic pregnancy. Others also thought that the IUD may cause inconveniences during sexual intercourse, pain for the male partner, as the strings are likely to hurt his penis, and that using the method creates pain and discomfort for the woman during intercourse [23]. For implants, it may be due to the myth that implants result in complications in the arm in which they are inserted and that they usually travel from the site of insertion to other parts of the body. Implants are also believed to cause problems such as infection, blindness, cancer and birth defects [23].

Looking at the trend of the age at first reporting over the three-year period, it implies that the general age of first reporting of contraceptive among the women ranges between 15 and 29. This is a very sexually active age group and for this reason, women who were still in school, unemployed, not yet married or married but had attained their preferred number of children may therefore decide to use contraceptives to prevent unwanted pregnancies. The 2010 Ghana Population and Housing Census acknowledged the fact that the age at first sexual intercourse in the country is 16 years [14]. It was therefore not surprising that the age at first reporting of contraceptive use for some women was 15 years.

In this study, DMPA offered the highest couple-years of protection against unwanted pregnancies over the three-year period, unlike male condom which offered the least. This is quite understandable since DMPA was most preferred to male condoms by the women as found by the study. This implies that DMPA provided the safest and longest protection for women or couples during the period. A similar observation was made by Okpani and Kua [22] where DMPA offered the highest couple-years of protection against unwanted pregnancies among study participants.

## Conclusions

Our results showed that DMPA was the most preferred choice of contraceptive method among the women from 2011 to 2013, and offered the highest level of protection for women who utilised it over the study period. However, the male condom, Norigynon, IUD and pills had been consistently under-utilised by the women in the catchment area over the period. It is therefore imperative to promote the use of the male condom, Norigynon, and IUD and pills among women in Kasoa and its environs in order to improve upon their contraceptive use rate and in turn reduce unintended pregnancies. The myths about the various contraceptives particularly the under-utilised ones should be dispelled by health professionals during family planning programmes organised for women, and the facts made clear to them. This would help allay the fears or misconceptions that some women may have and help promote utilisation of those contraceptive methods.
